# Challenges of COVID-19

**DOI:** 10.1515/almed-2020-0105

**Published:** 2020-11-12

**Authors:** Ignacio López-Goñi

**Affiliations:** Department of Microbiology and Parasitology, University of Navarra, Pamplona, Spain

On January 30, 2020 a comment was published in *Nature* on the amount of articles that had been published to date in relation to the novel coronavirus detected in China—more than 50 papers in less than a month. Quite a feat! [1]. In mid October, more than 60,000 articles on the virus SARS-CoV-2 or COVID-19 were indexed in PubMed. More articles have appeared on the novel coronavirus in nine months than on other diseases in decades. Such is the flood of scientific information available that it is impossible to thoroughly evaluate and study it. In the literature, we can find fake, ill-intentioned articles that contain misleading claims that promote the spread of fake news. However, the answer to many of the questions raised and uncertainties caused by this virus may lie in this huge amount of information. After a literature review, we retrieved some articles of acceptable quality that shed light on or suggest new ways to fight the pandemic in such relevant aspects as disease transfer, diagnosis, immunity, or the similarities of SARS-CoV-2 with other respiratory infectious agents.

The role of fomites, small droplets or aerosols in SARS-CoV-2 dissemination is still a matter of debate. SARS-CoV-2 does not seem to spread as easily as the measles virus, the most contagious transmissible virus known. Nevertheless, we are certainly facing a respiratory transmission disease. The difference between transmission by droplets or aerosols is an artificial, human-made classification. Vectors of transmission range from large droplets to tiny aerosols, and the physical and chemical properties of the viral particle determine how the disease spreads. Virus-laden droplets (larger than 100 micras) can contaminate surfaces located within a distance of 2 m in a few seconds. Physical distancing and hand hygiene reduce exposure to these droplets. In contrast, virus-laden aerosols (smaller than 100 micras) can stay in the air for several hours and be inhaled by subjects. It can be compared with tobacco smoke. Aerosols can travel more than 2 m and concentrate in indoor, poorly-ventilated spaces, thereby giving rise to superspreading events. A lower risk of transmission than expected has been observed in studies based on SARS-CoV-2 RNA detection and Vero E6 cell culture carried out to determine the presence of the virus on surfaces [2]. On the other hand, there is overwhelming evidence that the main route of transmission of COVID-19 is by inhalation of the virus [3]. A patient with COVID-19 can expel virus-containing fomites and aerosols during breathing and speaking. Therefore, added to recommendations on the use of masks, social distancing and hand hygiene to prevent disease transmission, it is essential to increase outdoor activities and reduce or avoid enclosed, poorly-ventilated, crowded spaces where people are speaking.

Diagnostics based on CRISPR genetics have revolutionized the detection of viral and bacterial pathogens. An example is the SHERLOCK system (**s**pecific **h**igh-sensitivity **e**nzymatic **r**eporter un**lock**ing) for specific ARN/ADN detection at attomolar concentrations [4]. A new, faster method based on SHERLOCK technology and isothermal amplification was developed recently. This system is claimed to detect the virus in less than 1 h, with a sensitivity and specificity comparable to that of RT-qPCR, but with reduced dependence on equipment [5]. This technique combines a simple magnetic viral ARN extraction technique with isothermal amplification at 60 °C and CRISPR-Cas12b detection. The limit of detection is 100 copies of viral genome input per reaction. This type of technologies can be used in small clinical laboratories and may revolutionize the diagnosis of SARS-CoV-2 infection in the coming months.

The role of community immunity, which occurs when a sufficient percentage of the population has become immune to the disease, as a strategy to prevent the spread of the pandemic has been the subject of a heated debate. Another controversial issue is whether previous exposure to the other human coronaviruses that cause common cold (HCoV-OC43, HCoV-HKU1, HCoV-229E, and HCoV-NL63) provides some immunity to SARS-CoV-2. Evidence has been provided recently on the presence of CD4+ memory T cells that are able to cross-react against SARS-CoV-2 in subjects not exposed to this virus. Lipsitch et al. [6] have described four potential scenarios on the epidemiological impact that pre-existing cross-reactive immune memory may have at individual and group level. According to the authors, these scenarios are based on the effect of cross-reaction on SARS-CoV-2 replication in the upper airways and the lungs, virus infectivity and the severity of COVID-19. In summary, the authors suggest that pre-existing immune memory should be considered in models for predicting the evolution of the pandemic.

The scientific community is worried about the upcoming flu season overlapping with COVID-19 in winter. A “perfect storm” scenario may unfold if SARS-CoV-2 overlaps with other coronaviruses such as the flu, the respiratory syncytial virus, and other viruses that cause bronchiolitis and pneumonia, which frequently require hospitalization and may cause death in vulnerable groups. Vásquez-Hoyos et al. [7] recently published a study on the number of admissions of children with respiratory problems caused by viral infections to pediatric intensive care units (PICUS) in four countries in Latin America. The authors compared 2020 data to the previous two years. The results show a 78–92% reduction of PICU admissions due to infections by viruses other than SARS-CoV-2 during the COVID-19 pandemic. In the same line, the incidence of flu in Chile, Australia or South Africa in June and August 2020 – the flu season in the South – decreased with respect to the previous years [8]. Although other factors may have contributed to reduce the incidence of flu this season, it is very likely that the lockdown, mask use, hand hygiene, social distancing, the reduction of population movements, and the intensification of the seasonal flu vaccination campaign have played a relevant role. Although the course of the flu season in the North Hemisphere this winter is still uncertain, these data are encouraging and support that use of preventive measures is maintained.

As aforementioned, although the volume of scientific information available is overwhelming and difficult to grasp, once again, science has demonstrated that knowledge and cooperation are the best way to beat the pandemic. In conclusion, there is room for hope.
